# Association Between Particulate Matter Exposure and Preterm Birth in Women With Abnormal Preconception Thyrotropin Levels: Large Cohort Study

**DOI:** 10.2196/53879

**Published:** 2024-08-02

**Authors:** Ting Xu, Haobo Ni, Xiaoyan Cai, Tingting Dai, Lingxi Wang, Lina Xiao, Qinghui Zeng, Xiaolin Yu, Lu Han, Pi Guo

**Affiliations:** 1 Department of Preventive Medicine, Shantou University Medical Colleage, Shantou, China; 2 NHC Key Laboratory of Male Reproduction and Genetics, Guangdong Provincial Reproductive Science Institute, Guangdong Provincial Fertility Hospital, Guangzhou, China

**Keywords:** PM2.5, particulate matter with an aerodynamic diameter of ≤2.5 μm, thyroid stimulating hormone, preterm birth, cohort study, preconception

## Abstract

**Background:**

Prior research has linked exposure to particulate matter with an aerodynamic diameter of ≤2.5 μm (PM_2.5_) with preterm birth (PTB). However, the modulating effect of preconception thyroid stimulating hormone (TSH) levels on the relationship between PM_2.5_ exposure and PTB has not been investigated.

**Objective:**

This study aimed to assess whether preconception TSH levels modulate the impact of PM_2.5_ exposure on PTB.

**Methods:**

This cohort study was conducted in Guangdong, China, as a part of the National Free Pre-Pregnancy Checkups Project. PM_2.5_ exposure was estimated by using the inverse distance weighting method. To investigate the moderating effects of TSH levels on trimester-specific PM_2.5_ exposure and PTB, we used the Cox proportional hazards model. Additionally, to identify the susceptible exposure windows for weekly specific PM_2.5_ exposure and PTB, we built distributed lag models incorporating Cox proportional hazards models.

**Results:**

A total of 633,516 women who delivered between January 1, 2014, to December 31, 2019, were included. In total, 34,081 (5.4%) of them had abnormal preconception TSH levels. During the entire pregnancy, each 10-μg/m^3^ increase in PM_2.5_ was linked to elevated risks of PTB (hazard ratio [HR] 1.559, 95% CI 1.390‐1.748), early PTB (HR 1.559, 95% CI 1.227‐1.980), and late PTB (HR 1.571, 95% CI 1.379‐1.791) among women with abnormal TSH levels. For women with normal preconception TSH levels, PM_2.5_ exposure during the entire pregnancy was positively associated with the risk of PTB (HR 1.345, 95% CI 1.307‐1.385), early PTB (HR 1.203, 95% CI 1.126‐1.285), and late PTB (HR 1.386, 95% CI 1.342‐1432). The critical susceptible exposure windows were the 3rd-13th and 28th-35th gestational weeks for women with abnormal preconception TSH levels, compared to the 1st-13th and 21st-35th gestational weeks for those with normal preconception TSH levels.

**Conclusions:**

PM_2.5_ exposure was linked with a higher PTB risk, particularly in women with abnormal preconception TSH levels. PM_2.5_ exposure appears to have a greater effect on pregnant women who are in the early or late stages of pregnancy.

## Introduction

With the decline of fertility, maternal and newborn health is receiving more attention. Preterm birth (PTB; birth at less than 37 complete gestational weeks), as a common adverse birth outcome, not only correlates with infant morbidity and mortality but also is a primary cause of death of children aged younger than 5 years [1-3]. According to the World Health Organization, there were approximately 13.4 million cases of PTB worldwide in 2020 [4]. PTB has been reported to have adverse effects on subsequent physical and cognitive development, placing a heavy burden on families, health systems, and socioeconomics [5,6]. Identifying the risk factors for PTB is an urgent issue, as it can help to prevent PTB.

Epidemiological studies have indicated that air pollution during pregnancy, especially involving particulate matter with an aerodynamic diameter of ≤2.5 μm (PM_2.5_), is a risk factor for PTB [7,8]. The levels of susceptibility of pregnant women to air pollution may vary depending on their physiological status [9]. For example, previous studies have shown that women with preexisting health problems such as diabetes and chronic hypertension are more sensitive to the detrimental consequence of air pollution [10]. In particular, maternal thyroid function could modulate the relationship of air pollution with birth outcomes. Pregnant women often experience thyroid dysfunction, which can increase the risks of PTB, spontaneous abortion, and other adverse birth outcomes [11]. Previous studies have found that prepregnancy thyroid disease might result in adverse birth outcomes, such as congenital heart defects, by modulating the effect of air pollution [12].

Thyroid stimulating hormone (TSH) is a crucial indicator of thyroid function. According to a nationwide cross-sectional study in China, the prevalence of abnormal thyroid stimulating levels has been estimated to be about 15.33% [13]. However, although the prevalence of abnormal TSH levels remains high and exerts great influence on individuals and society, studies on women who have abnormal TSH levels are limited. A study by Arbib et al [14] has revealed that abnormal TSH levels during early pregnancy are a risk factor for PTB and placental abruption. The impact of preconception TSH levels on pregnant women needs to be explored to encourage those women to adopt appropriate health care measures during pregnancy. Unfortunately, the pregnancy outcomes of women with abnormal prepregnancy TSH levels do not receive the attention they deserve. Only several studies have shown that women with lower or higher preconception TSH levels had increased risks of PTB [15,16]. Identifying the modulating effects of TSH on the relationship between air pollution and PTB is of great significance, as it can help to identify susceptible people and contribute to providing recommendations for mitigating the risks of PTB. Although the mechanism of PTB is complicated, it is widely recognized that protecting the vulnerable population to prevent the occurrence of PTB is an effective and feasible strategy.

The association between PM_2.5_ and PTB has been the subject of numerous studies [17-22]. However, to the best of our knowledge, there has been no study on whether maternal TSH levels could modulate the relationships of PM_2.5_ with PTB. Besides, most previous studies on air pollution and birth outcomes attempted to identify susceptible windows using time periods such as trimesters or gestational months [23-25]. However, a previous study [26] indicated that trimester-specific association might ignore the potential windows that span multiple trimesters and produce biased estimates.

Recently, a growing number of studies have recommended the use of more precise time windows, such as weeks, to identify the critical susceptible windows for health effects from atmospheric pollutants more accurately [26-28]. Identifying susceptible windows may help to explore the potential mechanism and provide guidance for the prenatal care of pregnant women. Nevertheless, no study has explored the susceptible windows for PTB from PM_2.5_ exposure at a weekly scale among women with abnormal preconception TSH levels.

In this study, we explored the modulating effects of TSH on the PM_2.5_-PTB association based on a large population-based cohort, which would provide a scientific basis for using preconception TSH levels among reproductive women as indicators for early intervention to prevent adverse birth outcomes. Our study had the following objectives: (1) to quantify and differentiate the effects of trimester-specific PM_2.5_ exposure on PTB and on PTB subtypes among women with different preconception TSH levels and (2) to explore the susceptible windows at weekly scales.

## Methods

### Study Population

We obtained data from the National Free Pre-Pregnancy Checkups Project (NFPCP), which aimed to offer free prepregnancy health examination and counseling to couples of reproductive ages who intended to become pregnant within the following 6 months. After prepregnancy examination, trained local health professionals followed up these couples through face-to-face or telephone interview to check on pregnancy status every 3 months for up to 1 year. If pregnant, health personnel would follow up with these participants on their lifestyle in the early stage of pregnancy and pregnancy outcomes after delivery. If pregnancy was not achieved within that year, follow-up was discontinued. Detailed information about how this project was designed, organized, and implemented can be found in previous studies [29-31].

**Figure 1.**
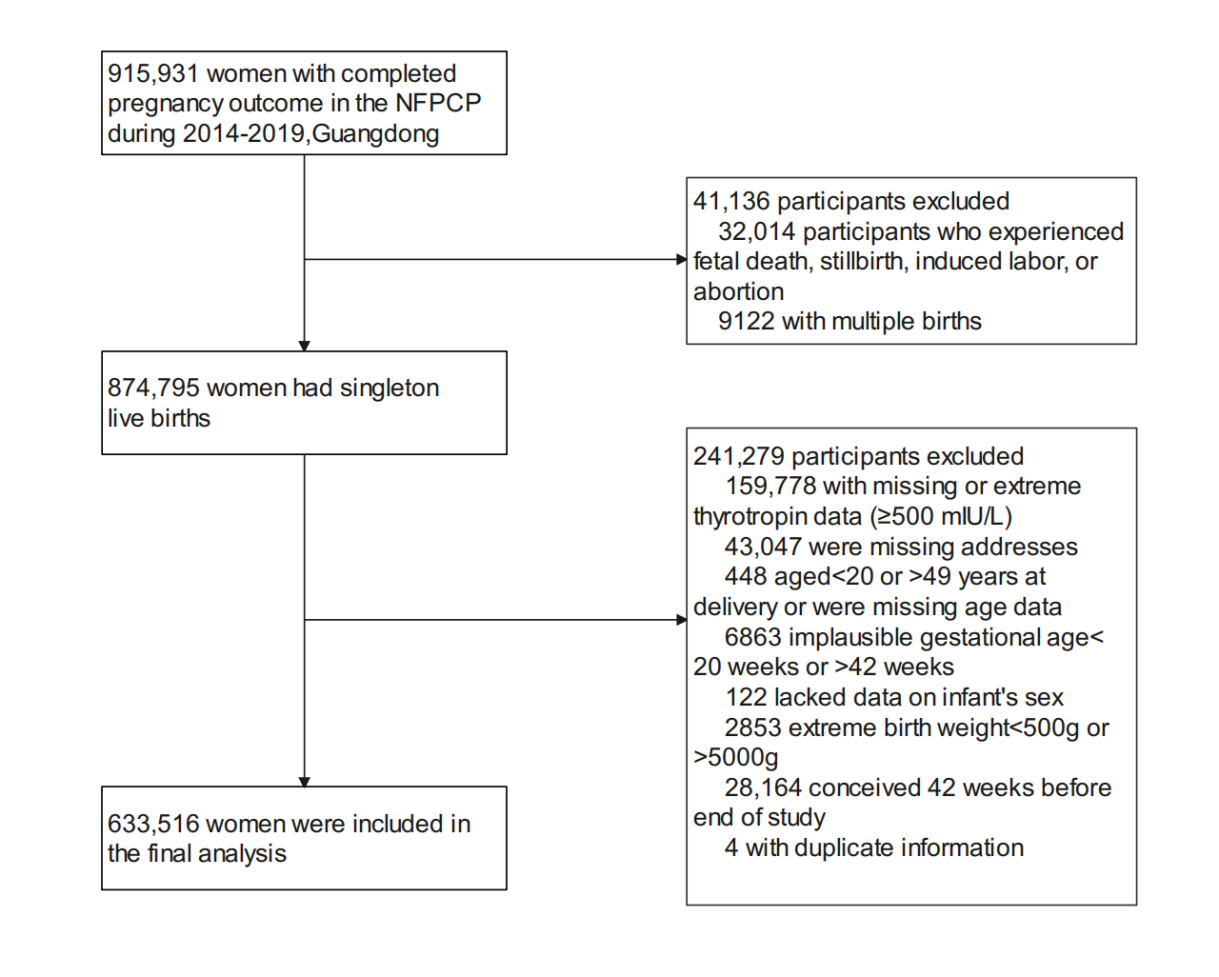
Flowchart of this study’s population based on the NFPCP. NFPCP: National Free Pre-Pregnancy Checkups Project.

The exclusion criteria for this cohort study are illustrated in Figure 1. Initially, we included 915,931 women from the NFPCP in Guangdong, China, who gave birth from January 1, 2014, to December 31, 2019, and had complete pregnancy outcomes. Then, we excluded 32,014 cases of fetal death, stillbirth, induced labor, or abortion; 9122 cases with multiple births; 159,778 cases with missing or extreme thyrotropin values (TSH>500 mIU/L); 43,047 cases with missing residential addresses; 448 cases of women with missing age data or women who were not of reproductive age (<20 or >49 years); 6863 cases with a gestational age of less than 20 weeks or greater than 42 weeks; 122 cases that lacked data on the infant’s sex; and 2853 cases of women who gave birth to babies with extreme birth weights (<500 g or >5000 g). Then, women who conceived 42 weeks before this study ended (December 31, 2019; n=28,164) were excluded, with the aim of avoiding fixed cohort bias [32]. In addition, 4 cases with duplicate information were excluded. Eventually, this study included 633,516 women who delivered living singletons.

### Outcome

PTB, defined as delivery at a gestational age of earlier than 37 weeks [33], was the primary outcome. In addition, we further divided PTB into 2 categories: early PTB (EPTB; 20‐33 wk of gestation) and late PTB (LPTB; 34‐36 wk of gestation) [34]. We determined the gestational age of the mother by taking into account the first day of her last menstrual period along with the delivery date.

### Measurement and Group Classification of TSH

In our study, serum TSH levels were assessed during the preconception period. Blood samples were collected from participants at the preconception examination, which occurred within 6 months before pregnancy. The serum TSH concentration was measured using an electrochemiluminescence immunoassay and a TSH detection kit.

Due to the lack of authoritative guidelines on the reference range of TSH among prepregnancy women, a population-based reference range of TSH was established from the current cohort. Based on previous studies [15,16], the reference population of 311,089 women was selected according to the following criteria: no previous adverse pregnancy outcomes; no history of thyroid diseases, anemia, high blood pressure, or diabetes mellitus; not taking oral contraceptives in the past; not consuming alcohol or cigarettes; having a normal BMI (18.5‐24 kg/m^2^); giving birth to healthy infants. The 2.5th and 97.5th percentiles for TSH levels were 0.10 mIU/L and 4.11 mIU/L, respectively. The reference range of TSH for women who planned to become pregnant in this cohort was 0.10‐4.11 mIU/L.

According to the reference range of TSH, we divided all participants into 2 groups: the normal TSH group (TSH 0.10‐4.11 mIU/L) and the abnormal TSH group (TSH <0.10 or TSH >4.11 mIU/L).

### Exposure Assessment

We collected daily (24 h) concentration data of PM_2.5_ (μg/m^3^) from air pollution monitoring stations located within Guangdong province from the China National Environmental Monitoring Centre. Daily concentrations of SO_2_ (μg/m^3^), NO_2_ (μg/m^3^), and O_3_ (μg/m^3^) were also obtained from the same monitoring stations for adjustment of concurrent exposure to gaseous pollutants.

We geocoded the residential addresses of all pregnant women enrolled in this study to longitude and latitude coordinates using the Gaode Maps open platform. Then, the air pollution exposure of each participant was estimated by using the inverse distance weighting (IDW) method, which has been extensively used in epidemiological research [1-3,21,25,27,35]. Previous studies [36,37] have reported that the health effects of environmental exposure on fetal development varied by trimesters of pregnancy. Therefore, we estimated the exposure to air pollutants for all pregnant women during the first trimester (0‐13 wk of gestation), the second trimester (14‐26 wk of gestation), and the third trimester (27 wk of gestation to delivery), as well as the entire pregnancy. To investigate more refined critical exposure windows during pregnancy, we estimated individual exposure to air pollutants during each gestational week. The time frame of exposure for pregnant women during the entire pregnancy was shown in Multimedia Appendix 1.

Furthermore, to account for potential meteorological confounding factors, we obtained daily mean ambient temperature (℃) and relative humidity (%) from meteorological monitoring stations in Guangdong province via the China Meteorological Data Sharing System. A similar approach for meteorological factors such as air pollution exposure was adopted.

### Statistical Analysis

Numbers (percentages) were used to express participants’ baseline characteristics according to TSH levels. Using Pearson correlation, we examined the correlation between air pollutants and meteorological factors in Guangdong from 2014 to 2019.

To evaluate the effect of PM_2.5_ exposure throughout the whole pregnancy as well as during each trimester on PTB and PTB subtypes, Cox proportion hazard models were constructed by treating PTB as the outcome and gestational week as the time scale. The effect of prenatal PM_2.5_ exposure on PTB and its subtypes was evaluated separately in women with normal and abnormal preconception TSH levels. The effects were expressed as the hazard ratios (HR) for each 10-μg/m^3^ rise in PM_2.5_ concentration during different pregnancy periods. According to previous studies [38], we adjusted for maternal age (<25, 25‐34, or ≥35 years), delivery mode (vaginal delivery or cesarean delivery), prepregnancy BMI (<18.5, 18.5‐24, 24‐28, or >28 kg/m^2^), maternal smoking (yes or no), maternal drinking (yes or no), the season of delivery (spring, summer, autumn, or winter), and infant’s sex (male or female). In addition, based on previous studies [38,39], natural splines with *df* of 6 and 3 were used to adjust the nonlinearity of ambient temperature and relative humidity, respectively. Moreover, we built distributed lag models incorporating Cox proportional hazards models with adjustments for the aforementioned covariates to identify more refined critical susceptible exposure windows [27,40]. This model could explore the exposure-response association and lag-response association simultaneously based on a “cross-basis” function. In the distributed lag models of this study, the exposure-response association was assumed to vary smoothly across gestational weeks. The lag distribution of PM_2.5_ was modeled as natural cubic splines with optimal *df* of 4 based on the minimum of the Akaike Information Criterion by varying *df* from 3 to 10. Additionally, the maximum lag range was set at week 36, since term birth was censored at week 37. The analysis was performed for EPTB and LPTB as we did for overall PTB, except that the exposure period was confined to gestational weeks 1 to 33 for early PTB.

Several sensitivity analyses were conducted. First, we changed the *df* for mean temperature from 5 to 7 and the *df* for relative humidity from 2 to 4. Second, all participants were categorized into the abnormal TSH and normal TSH groups based on the reference range of TSH (0.1‐4 mIU/L) for mothers during early pregnancy recommended by the American Thyroid Association [41], as some researchers [42] suggested that pregnant women in early pregnancy and nonpregnant women have similar reference ranges for TSH. Third, we excluded participants whose baseline characteristics were missing and then conducted the analysis. Last, considering the potential confounding effect of other air pollutants (NO_2_, SO_2_, or O_3_), 2-pollutant models were constructed to explore the relationship between atmospheric pollutants and PTB and PTB subtypes.

Statistical analysis was carried out using R (version 4.2.1; R Foundation for Statistical Computing). All statistical tests were 2-sided, and all statistics were considered significant when the *P* value was lower than .05.

### Ethical Considerations

This study was approved by the Medical Ethics Committee of the Guangdong Provincial Reproductive Science Institute (ID of the ethics approval: 202216). This study was in line with the Helsinki ethical guidelines. Written informed consent was obtained from all participants. Before recruitment, each participant provided written informed consent. This study used anonymized data in order to protect the privacy of participants, and no individually identifiable information was available.

## Results

In total, 633,516 women were eventually included in this study. A summary of all participant’s demographic characteristics is presented in Table 1. Among them, 599,435 (94.6%) women had normal preconception TSH levels, while 34,081 (5.4%) women had abnormal preconception TSH levels.

**Figure 2.**
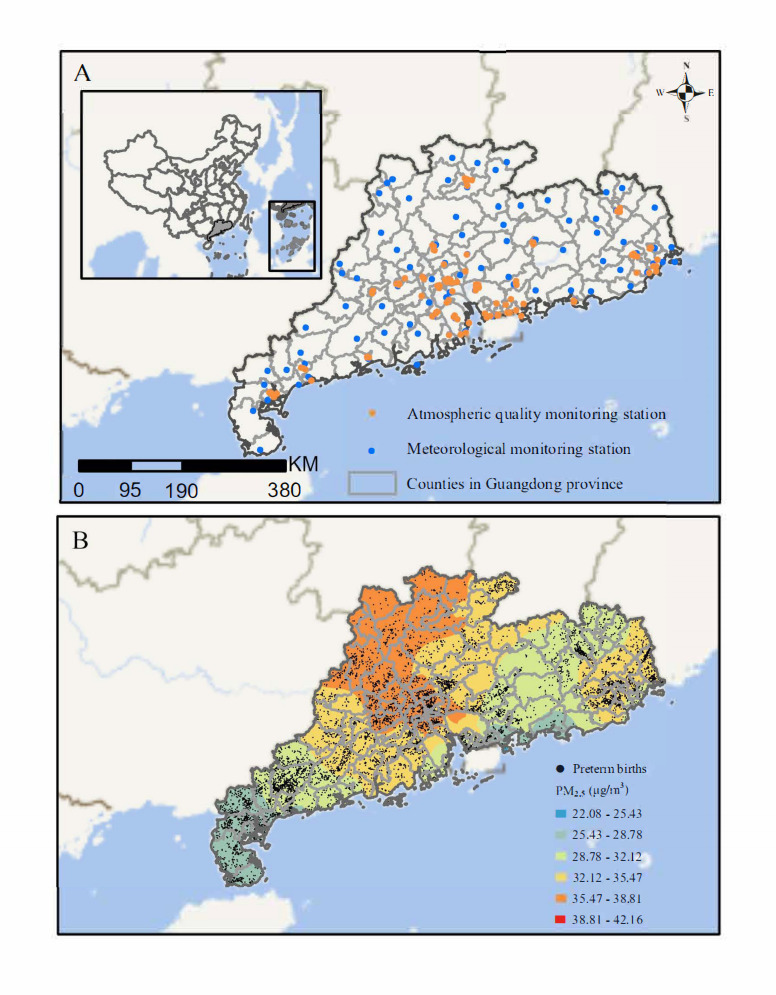
Geographical distribution of air pollution monitoring stations, meteorological monitoring stations, and preterm birth and spatial variation of PM_2.5_ concentration. (**A**) Spatial distribution of air pollution and meteorological monitoring stations. (**B**) Geographical distribution of preterm birth and spatial variation of PM_2.5_ concentration, as estimated by using the IDW spatial interpolation algorithm, from 2014 to 2019. E: east; IDW: inverse distance weighting; KM: kilometer; N: north; PM_2.5_: particulate matter with an aerodynamic diameter of ≤2.5 μm; S: south; W: west.

Figure 2 shows the spatial distribution of air pollution monitoring stations, meteorological stations, PTB, and PM_2.5_ exposure in Guangdong, China. The average exposure levels of PM_2.5_ throughout pregnancy were 32.5 (SD 5.8) μg/m^3^ (Table 2). Throughout pregnancy, the average temperature for all participants was 22.9 (SD 1.4) ℃, and the relative humidity was 79.9% (SD 3%). The correlation between weather conditions and exposure to air pollutants is presented in Multimedia Appendix 2. More specifically, PM_2.5_ exposure was positively linked to NO_2_ and SO_2_ (Pearson *r* 0.552 to 0.641) and negatively correlated with O_3_, temperature, and relative humidity (Pearson *r* −0.413 to −0.273) throughout pregnancy.

**Table 1.** Characteristics of study population.

Characteristics		Normal TSH^a^ level			Abnormal TSH level		
		Preterm births (n=22,351), n (%)	Term births (n=577,084), n (%)	Total births (n=599,435), n (%)	Preterm births (n=1335), n (%)	Term births (n=32,746), n (%)	Total births (n=34,081), n (%)
**Maternal age (years)**							
	<25	8486 (38)	192,117 (33.3)	200,603 (33.5)	484 (36.3)	10,237 (31.3)	10,721 (31.5)
	25‐34	12,528 (56.1)	349,170 (60.5)	361,698 (60.3)	746 (55.9)	20,196 (61.7)	20,942 (61.5)
	>35	1337 (5.9)	35,797 (6.2)	37,134 (6.2)	105 (7.8)	2313 (7)	2418 (7)
**Prepregnancy BMI** **(kg/m**^**2**^**)**							
	<18.5	4794 (21.5)	114,416 (19.8)	119,210 (19.9)	346 (25.9)	8042 (24.6)	8388 (24.6)
	18.5‐24	14,780 (66.1)	384,525 (66.6)	399,305 (66.6)	837 (62.7)	20,977 (64.1)	21,814 (64)
	24‐28	2147 (9.6)	61,907 (10.7)	64,054 (10.7)	112 (8.4)	2887 (8.8)	2999 (8.8)
	>28	495 (2.2)	13,057 (2.3)	13,552 (2.3)	28 (2.1)	588 (1.8)	616 (1.8)
	Missing data	135 (0.6)	3179 (0.5)	3314 (0.5)	12 (0.9)	252 (0.7)	264 (0.8)
**Maternal smoking during pregnancy**							
	Yes	52 (0.2)	1374 (0.2)	1426 (0.2)	2 (0.2)	103 (0.3)	105 (0.3)
	No	22,197 (99.3)	573,321 (99.4)	595,518 (99.4)	1327 (99.4)	32,523 (99.3)	33,850 (99.3)
	Missing data	102 (0.5)	2389 (0.4)	2491 (0.42)	6 (0.4)	120 (0.4)	126 (0.4)
**Maternal drinking during pregnancy**							
	Yes	1388 (6.2)	36,001 (6.2)	37,389 (6.2)	102 (7.7)	2526 (7.7)	2628 (7.7)
	No	20,776 (93)	537,184 (93.1)	557,960 (93.1)	1218 (91.2)	30,040 (91.7)	31,258 (91.7)
	Missing data	187 (0.8)	3899 (00.7)	4086 (0.7)	15 (1.1)	180 (0.6)	195 (0.6)
**Mode of delivery**							
	Vaginal	18,089 (80.9)	454,502 (78.8)	472,591(78.8)	999 (74.8)	24,421 (74.6)	25,420 (74.6)
	Cesarean	4262 (19.1)	122,582 (21.2)	126,844 (21.2)	336 (25.2)	8325 (25.4)	8681 (25.4)
**Infant’s sex**							
	Male	12,315 (55.1)	302,807 (52.5)	315,122 (52.6)	722 (54.1)	16,525 (50.5)	17,247 (50.6)
	Female	10,036 (44.9)	274,277 (47.5)	284,313 (47.4)	613 (45.9)	16,221 (49.5)	16,834 (49.4)
**Season of delivery**							
	Spring	5040 (22.5)	134,507 (23.3)	139,547 (23.3)	306 (22.9)	8001 (24.4)	8307 (24.4)
	Summer	5511 (24.7)	124,202 (21.5)	129,713 (21.6)	326 (24.4)	6969 (21.3)	7295 (21.4)
	Autumn	6183 (27.7)	159,983 (27.7)	166,166 (27.7)	351 (26.3)	8661 (26.5)	9012 (26.4)
	Winter	5617 (25.1)	158,392 (27.5)	164,009 (27.4)	352 (26.4)	9115 (27.8)	9467 (27.8)

a TSH: thyroid stimulating hormone.

**Table 2.** Summary of air pollutant exposure and weather conditions during the pregnancies of all participants.

Variables		Mean (SD)	Min^a^	Max^b^	25th^c^	50th^d^	75th^e^
**First trimester**							
	PM_2.5_^f^ (μg/m^3^)	32.6 (10.1)	9	74	25.2	32	38.8
	O_3_ (μg/m^3^)	54.4 (10.8)	8.6	93.7	47.6	54.8	62.3
	SO_2_ (μg/m^3^)	12.5 (4.5)	3.8	35.7	9.2	11.6	14.6
	NO_2_ (μg/m^3^)	26.7 (12.5)	5.8	82.9	16.1	24.2	34.9
	Temperature (℃)	22.9 (1.4)	13.3	27.7	22.1	23	23.7
	Relative humidity (%)	79.8 (3)	66.9	89.6	77.7	80	82.1
**Second trimester**							
	PM_2.5_ (μg/m^3^)	31.8 (10.2)	9.1	72.3	24	31.1	38.2
	O_3_ (μg/m^3^)	55.2 (10.6)	10.9	94.2	48.6	55.3	62.9
	SO_2_ (μg/m^3^)	12.2 (4.2)	3.2	35.5	9.1	11.5	14.4
	NO_2_ (μg/m^3^)	26.3 (12.7)	5.5	82.9	15.6	23.5	34.4
	Temperature (℃)	23 (1.4)	13.4	27.6	22.2	23.1	23.8
	Relative humidity (%)	80 (3)	68.2	89.6	77.9	80.2	82.2
**Third trimester**							
	PM_2.5_ (μg/m^3^)	33.1 (10.4)	7.7	82	25.4	32.7	39.9
	O_3_ (μg/m^3^)	56.3 (10.8)	11.5	110.2	49.6	56.7	64.1
	SO_2_ (μg/m^3^)	12.2 (4.2)	2.8	35.9	9.1	11.5	14.3
	NO_2_ (μg/m^3^)	27 (12.9)	5.8	93.1	16.3	24.3	35.2
	Temperature (℃)	22.9 (1.4)	11.9	29.3	22.1	23	23.8
	Relative humidity (%)	79.9 (3)	61	89.4	77.8	80.1	82.1
**Entire pregnancy**							
	PM_2.5_ (μg/m^3^)	32.5 (5.8)	16	59.7	28.1	32.1	36.4
	O_3_ (μg/m^3^)	55.3 (7.7)	15.1	85.2	50.4	55.9	60.8
	SO_2_ (μg/m^3^)	12.3 (3.8)	4.2	30.6	9.7	11.7	13.9
	NO_2_ (μg/m^3^)	26.6 (11.5)	8.4	69.1	16	23.7	35
	Temperature (℃)	22.9 (0.9)	18.3	25.8	22.4	23.1	23.6
	Relative humidity (%)	79.9 (2.8)	69.5	88.3	77.9	80	82.1

a Min: minimum.

b Max: maximum.

c 25th: 25th percentile.

d 50th: 50th percentile.

e 75th: 75th percentile.

f PM_2.5_: particulate matter with an aerodynamic diameter of ≤2.5 μm.

The effect of PM_2.5_ exposure at various stages of pregnancy on PTB and its subtypes is shown in Table 3. For women with normal and abnormal prepregnancy TSH levels, positive associations were observed between PM_2.5_ exposure and the risks of PTB at each trimester and throughout pregnancy. Notably, the effects were greater among women with abnormal preconception TSH levels. For instance, for women with abnormal and normal preconception TSH levels, each 10-μg/m^3^ rise in PM_2.5_ throughout pregnancy was linked to a 55.9% (HR 1.559, 95% CI 1.390‐1.748) and 34.5% (HR 1.345, 95% CI 1.307‐1.385) increase in risks of PTB, respectively. In addition, Table 3 also presents the relationship between PM_2.5_ exposure and risks of EPTB, as well as LPTB. The results for PTB subtypes were approximately consistent with those for all PTBs. Generally, the associations with LPTB were higher than those with EPTB. For EPTB, we observed significant associations among women with abnormal TSH levels (HR 1.559, 95% CI 1.227‐1.980) and women with normal TSH levels (HR 1.203, 95% CI 1.126‐1.285) with each 10-μg/m^3^
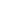
increase of PM_2.5_ during the entire pregnancy. As for LPTB, significant associations were also observed, with the corresponding HRs being 1.571 (95% CI 1.379‐1.791) and 1.386 (95% CI 1.342‐1.432), respectively.

**Figure 3.**
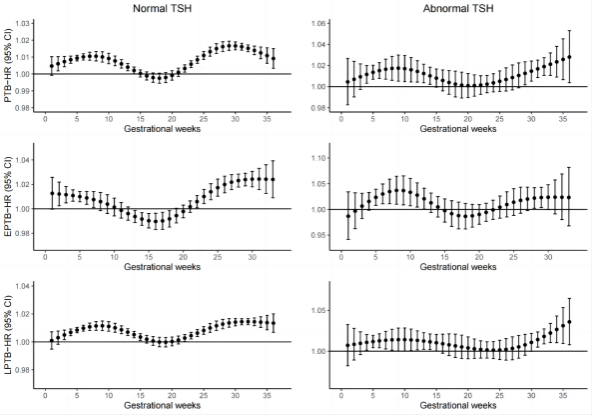
Associations between weekly specific PM_2.5_ exposure and risk of PTB according to maternal prepregnancy status of TSH. DLMs incorporating Cox proportional hazard models were used to calculate the HR (95% CI) for each 10-μg/m^3^ increment in PM_2.5_ during gestation weeks. The model was adjusted for maternal age, delivery mode, prepregnancy BMI, maternal smoking, maternal drinking, delivery season, and infant’s sex, as well as for mean ambient temperature and relative humidity during the pregnancy with natural cubic splines of 6 and 3 *df*, respectively. DLM: distributed lag model; EPTB: early preterm birth; HR: hazard ratio; LPTB: late preterm birth; PM_2.5_: particulate matter with an aerodynamic diameter of ≤2.5 μm; PTB: preterm birth; TSH: thyroid stimulating hormone.

Figure 3 shows the associations between weekly specific PM_2.5_ exposure and PTB. For women with abnormal preconception TSH levels, PM_2.5_ exposure between the 3rd and 13th weeks of pregnancy, along with the 28th to 35th weeks of pregnancy, were linked to elevated risks of PTB, with the strongest association observed during the 35th week (HR 1.028, 95% CI 1.004‐1.053). For women with normal preconception TSH levels, the exposure windows were the 1st-13th and the 21st-35th weeks of gestation, with the peak association occurring at the 29th week (HR 1.017, 95% CI 1.014‐1.018). There were similar identified exposure windows and magnitudes of associations between PM_2.5_ exposure and EPTB, LPTB, and overall PTB.

**Table 3.** Associations between trimester-specific PM_2.5_^a^ exposure and risk of PTB^b^ according to maternal prepregnancy status of TSH^c^^,^^d^.

PTB types and gestational period		Normal TSH, HR^e^^,^^f^ (95% CI)	Abnormal TSH, HR (95% CI)
**All PTBs**			
	First trimester	1.111 (1.095‐1.127)	1.129 (1.064‐1.198)
	Second trimester	1.117 (1.098‐1.136)	1.270 (1.186‐1.360)
	Third trimester	1.044 (1.029‐1.060)	1.087 (1.0231.155)
	Entire pregnancy	1.345 (1.307‐1.385)	1.559 (1.390‐1.748)
**Early PTB**			
	First trimester	1.108 (1.072‐1.145)	1.102 (0.972‐1.248)
	Second trimester	1.034 (0.995‐1.075)	1.355 (1.175‐1.563)
	Third trimester	1.069 (1.030‐1.110)	1.109 (0.967‐1.272)
	Entire pregnancy	1.203 (1.126‐1.285)	1.559 (1.227‐1.980)
**Late PTB**			
	First trimester	1.113 (1.095‐1.131)	1.139 (1.065‐1.218)
	Second trimester	1.138 (1.117‐1.160)	1.249 (1.156‐1.351)
	Third trimester	1.039 (1.022‐1.056)	1.084 (1.013‐1.160)
	Entire pregnancy	1.386 (1.342‐1.432)	1.571 (1.379‐1.791)

a PM_2.5_: particulate matter with an aerodynamic diameter of 2.5 μm or less.

b PTB: preterm birth.

c TSH: thyroid stimulating hormone.

d Model was adjusted for maternal age, delivery mode, prepregnancy BMI, maternal smoking, maternal drinking, delivery season, and infant’s sex, as well as for mean ambient temperature and relative humidity during the pregnancy with natural cubic splines of 6 and 3 *df*, respectively.

e HR: hazard ratio.

f Hazard ratios are based on 10-μg/m3 increase in PM_2.5_ exposure.

**Figure 4.**
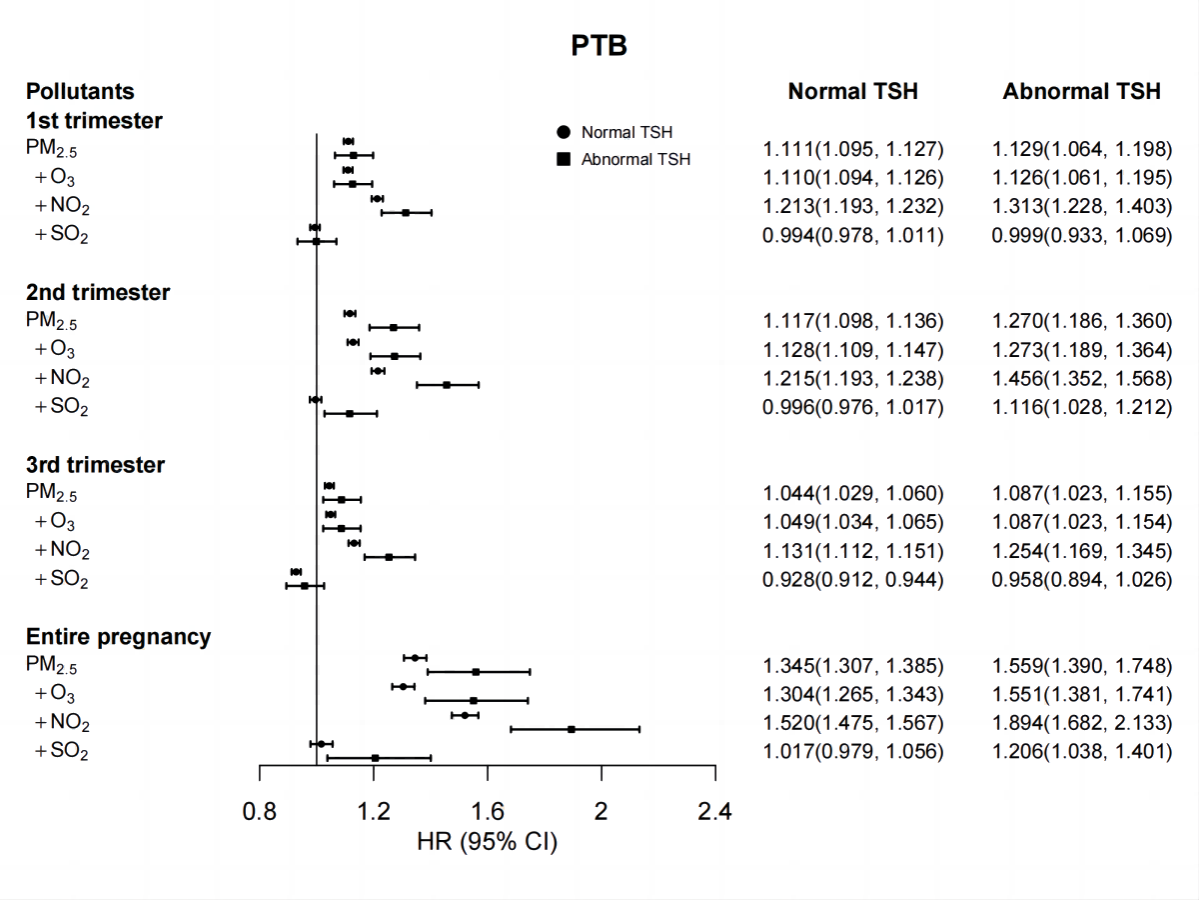
HR (95% CI) of PTB associated with each 10-μg/m^3^ increase in air pollutant concentration during the pregnancy in 2-pollutant models compared to single-pollutant models. Single-pollutant models were adjusted for maternal age, delivery mode, prepregnancy BMI, maternal smoking, maternal drinking, delivery season, and infant’s sex, as well as for mean ambient temperature and relative humidity during the pregnancy with natural cubic splines of 6 and 3 *df*, respectively. Further, 2-pollutant models were adjusted for the variable considered in single-pollutant models, in addition to including the air pollutant shown above. HR: hazard ratio; PM_2.5_: particulate matter with an aerodynamic diameter of ≤2.5 μm; PTB: preterm birth; TSH: thyroid stimulating hormone.

In sensitivity analyses, the results did not bring substantial changes. We obtained similar results when changing the *df* for temperature and relative humidity (Multimedia Appendix 3). Similar results were observed when using 0.10‐4.0 mIU/L as the reference range of preconception TSH (Multimedia Appendix 4). The results were similar when we excluded participants whose baseline characteristics were missing from the analysis (Multimedia Appendix 5). Furthermore, the results of the 2-pollutant models were found to be similar to those of the single-pollutant models (Figure 4, Multimedia Appendices 6 and 7). In general, we also observed positive associations between PM_2.5_ exposure and PTB in 2-pollutant models among women with abnormal preconception TSH levels and women with normal preconception TSH levels.

## Discussion

### Principal Findings

We investigated the associations between PM_2.5_ exposure and PTB in women with abnormal and normal preconception TSH levels. This study is the first to examine the modulating effect of preconception TSH levels on the association between PM_2.5_ exposure and PTB. According to our study, PM_2.5_ exposure throughout the entire pregnancy was linked to elevated risks of PTB in women with abnormal and normal preconception TSH levels. Our findings would provide important population-based evidence and a scientific basis for preconception care to further promote maternal and child health.

There is increasing research on the relationship between PM_2.5_ exposure and PTB. Among women with abnormal and normal preconception TSH levels, our study observed a positive association between PM_2.5_ and PTB, similar to previous studies [8,43,44]. For instance, Laurent et al [44] found that for every increase in the IQR of PM_2.5_ throughout the pregnancy, the risks of PTB increased by 13.3%. However, no significant association has been found in some studies [44-47]. Disagreement among studies may be due to differences in the study design, exposure levels to air pollutants, particulate matter components, as well as the sample size.

Additionally, in women with abnormal preconception TSH levels, stronger associations between PM_2.5_ exposure and PTB were found compared to women with normal preconception TSH levels. A previous study indicated that thyroid hormone might play a role in inflammation related to PTB [48]. In addition, some researchers found that PM_2.5_ exposure could contribute to inflammation [49,50]. We hypothesize that the exacerbated risk of PTB observed in women with thyroid dysfunction might be attributed to a higher vulnerability to the inflammatory and oxidative stress effects of PM_2.5_. The interrelationship between thyroid disorder and increased susceptibility to environmental pollutants could be explained by several mechanisms. First, thyroid dysfunction may impair the body’s antioxidant defenses, rendering individuals more susceptible to oxidative damage induced by PM_2.5_ [51]. Second, altered thyroid hormone levels can disrupt the immune system balance, potentially exacerbating the inflammatory response to air pollution [52]. Additionally, maternal thyroid disorder may increase the risk of gestational diabetes and hypertension, which are risk factors for PTB [53,54]. However, the underlying biological mechanisms of these associations remain unclear due to the lack of toxicological evidence. Further studies are warranted to explore how TSH levels modulate the effects of PM_2.5_ exposure on PTB.

Furthermore, our study also identified the sensitive exposure windows. We found that the sensitive windows might be the 3rd-13th and 28th-35th gestational weeks for women with abnormal preconception TSH levels. We also found that the sensitive windows might be the 1st-13th and the 21st-35th gestational weeks for women with normal preconception TSH levels. It appears that early pregnancy and late pregnancy might be the susceptible exposure windows. A previous study [55] suggested that PM_2.5_ exposure in late pregnancy may stimulate the release of cytokines that promote inflammation, thereby triggering PTB. Additionally, some studies have suggested [55,56] that early pregnancy might be the most sensitive window. However, there has been no consistent conclusion regarding the susceptible exposure window. Therefore, further studies are warranted to confirm our results.

Only a very small number of studies have explored the relationship between PM_2.5_ exposure and specific PTB subtypes. Our study found a stronger association between PM_2.5_ exposure during the entire pregnancy and LPTB than that between such PM_2.5_ exposure and EPTB in women with abnormal and normal preconception TSH levels. We speculate that the effects of air pollution may be “masked” by other risk factors such as intrauterine infections and nutritional deficiencies in infants born early preterm.

Our study results have important public health implications. Previous studies have noted that PTB could place significant burdens on families, health systems, and socioeconomics. To mitigate the risks of PTB, women with abnormal preconception TSH levels should take additional protective measures against air pollution, as they were identified as the susceptible population in this study. For women with abnormal preconception TSH levels, we recommend enhancing the education on reproductive knowledge and improving the accessibility of health care services.

Our study has several strengths. First of all, this study has a relatively large sample size, enabling sufficient statistical power to assess the modulating effect of TSH on the impact of PM_2.5_ exposure on PTB. Second, we explored more precise susceptible windows, which could help us to develop specific clinical and public health interventions to reduce the risks of PTB, as well as inform research on potential etiological mechanisms regarding the relationship between PM_2.5_ and PTB. Third, we investigated the relationships between PM_2.5_ exposure and PTB subtypes as well.

However, several limitations should be noted. First, we did not collect the TSH levels during pregnancy. Therefore, classification based on preconception TSH alone may not be adequate to accurately reflect the modulating effects of serum TSH levels on the association between PM_2.5_ and PTB. Second, couples who participated in the NFPCP were those who intended to become pregnant, which may have led to selection bias with regard to those with unplanned pregnancies. More specifically, participants who plan to become pregnant will be less exposed to other risk factors compared to those with unplanned pregnancies owing to them taking protective measures or developing a healthy lifestyle. Third, air pollution exposure for all participants was estimated based on the residential addresses using the IDW method, a common practice in environmental health research [21,25,27,35]. This method assumes spatial continuity, where PM_2.5_ concentrations at unmeasured locations are interpolated based on nearby monitoring station data, adhering to the principle that environmental measurements in closer proximity are more similar than those farther apart. While this approach requires assumptions about spatial distribution and participant mobility, the IDW method was used for its applicability in large-scale epidemiological studies where direct, individual-level exposure measurement is challenging. More advanced exposure assessment methods, such as land use regression or random forest, were not conducted in this study because we did not have research data such as land use information, which is often used in these exposure assessment methods. Future studies should consider more accurate exposure assessment methods. In addition, we did not consider the mobility of participants due to unavailable information on residential mobility. Therefore, exposure misclassification was possible in this study. Fourth, due to the lack of relevant information, some confounding factors, such as thyroid-related medications, were not adjusted in the model.

### Conclusion

In summary, PM_2.5_ exposure during pregnancy increased PTB risks for women with abnormal and normal preconception TSH levels. Additionally, stronger associations were found among women with abnormal preconception TSH levels. Moreover, women in early or late pregnancy appear to be more vulnerable to harmful effects from air pollution. Additional protective measures against air pollution, such as wearing masks and using air purifiers, should be taken, especially for pregnant women with abnormal preconception TSH levels.

## Supplementary material

10.2196/53879 Multimedia Appendix 1 The time frame of PM_2.5_ exposure for study participants during the entire pregnancy. PM_2.5_: particulate matter with an aerodynamic diameter of ≤2.5 μm.

10.2196/53879 Multimedia Appendix 2 Pearson correlation coefficients of air pollutants and meteorological variables during entire pregnancy.

10.2196/53879 Multimedia Appendix 3 Sensitivity analyses of associations between trimester-specific PM_2.5_ exposure and risk of PTB according to maternal prepregnancy status of TSH. PM_2.5_: particulate matter with an aerodynamic diameter of ≤2.5 μm; PTB: preterm birth; TSH: thyroid stimulating hormone.

10.2196/53879 Multimedia Appendix 4 Associations between trimester-specific PM_2.5_ exposure and risk of PTB according to maternal preconception TSH levels using 0.10-4.00 mIU/L as the reference range. PM_2.5_: particulate matter with an aerodynamic diameter of ≤2.5 μm; PTB: preterm birth; TSH: thyroid stimulating hormone.

10.2196/53879 Multimedia Appendix 5 Associations between trimester-specific PM_2.5_ exposure and risk of PTB according to maternal prepregnancy status of TSH, excluding participants missing baseline characteristics (N=624,349). PM_2.5_: particulate matter with an aerodynamic diameter of ≤2.5 μm; PTB: preterm birth; TSH: thyroid stimulating hormone.

10.2196/53879 Multimedia Appendix 6 HR (95% CI) of EPTB associated with each 10-μg/m^3^ increase in air pollutant concentration during the pregnancy in 2-pollutant models compared to single-pollutant models. Single-pollutant models were adjusted for maternal age, delivery mode, prepregnancy BMI, maternal smoking, maternal drinking, delivery season, and infant’s sex, as well as for mean ambient temperature and relative humidity during the pregnancy with natural cubic splines of 6 and 3 *df*, respectively. Further, 2-pollutant models were adjusted for the variable considered in single-pollutant models, in addition to including the air pollutant shown. HR: hazard ratio; EPTB: early preterm birth.

10.2196/53879 Multimedia Appendix 7 HR (95% CI) of LPTB associated with each 10-μg/m^3^ increase in air pollutant concentration during the pregnancy in 2-pollutant models compared to single-pollutant models. Single-pollutant models were adjusted for maternal age, delivery mode, prepregnancy BMI, maternal smoking, maternal drinking, delivery season, and infant’s sex, as well as for mean ambient temperature and relative humidity during the pregnancy with natural cubic splines of 6 and 3 *df*, respectively. Further, 2-pollutant models were adjusted for the variable considered in single-pollutant models, in addition to including the air pollutant shown above. HR: hazard ratio; LPTB: late preterm birth.

## Abbreviations

EPTB: early preterm birth
HR: hazard ratio
IDW: inverse distance weighting
LPTB: late preterm birth
NFPCP: National Free Pre-Pregnancy Checkups Project
PM_2.5_: particulate matter with an aerodynamic diameter of ≤2.5 μm
PTB: preterm birth
TSH: thyroid stimulating hormone
