# Supplementary figures and images for: Association Between Particulate Matter Exposure and Preterm Birth in Women With Abnormal Preconception Thyrotropin Levels: Large Cohort Study
Source: JMIR Public Health Surveill. 2024 Aug 2;10:e53879. doi: 10.2196/53879 (PMC11310741; doi:10.2196/53879)

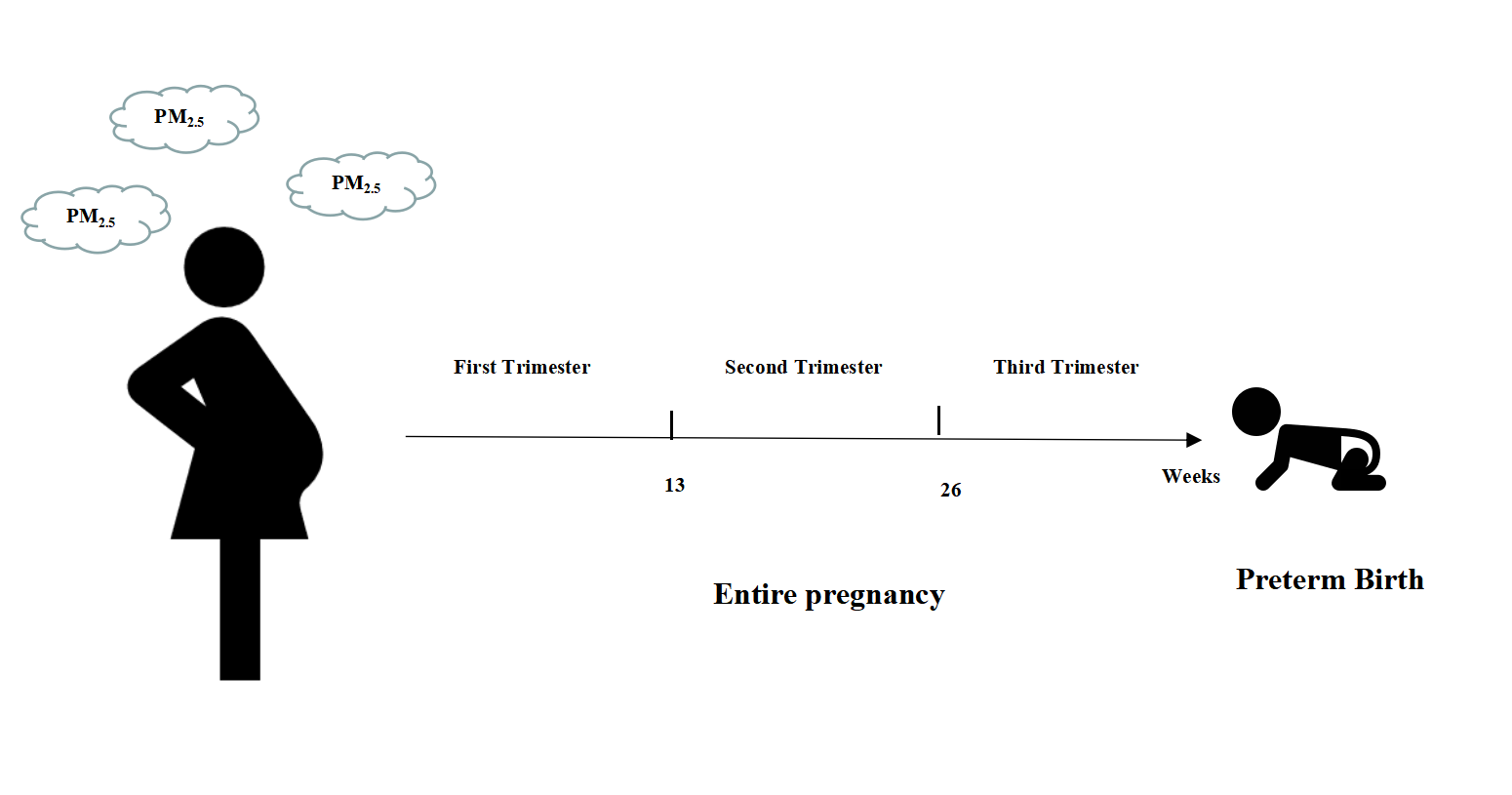

Supplement: Multimedia Appendix 1 [file publichealth-v10-e53879-s001.png]

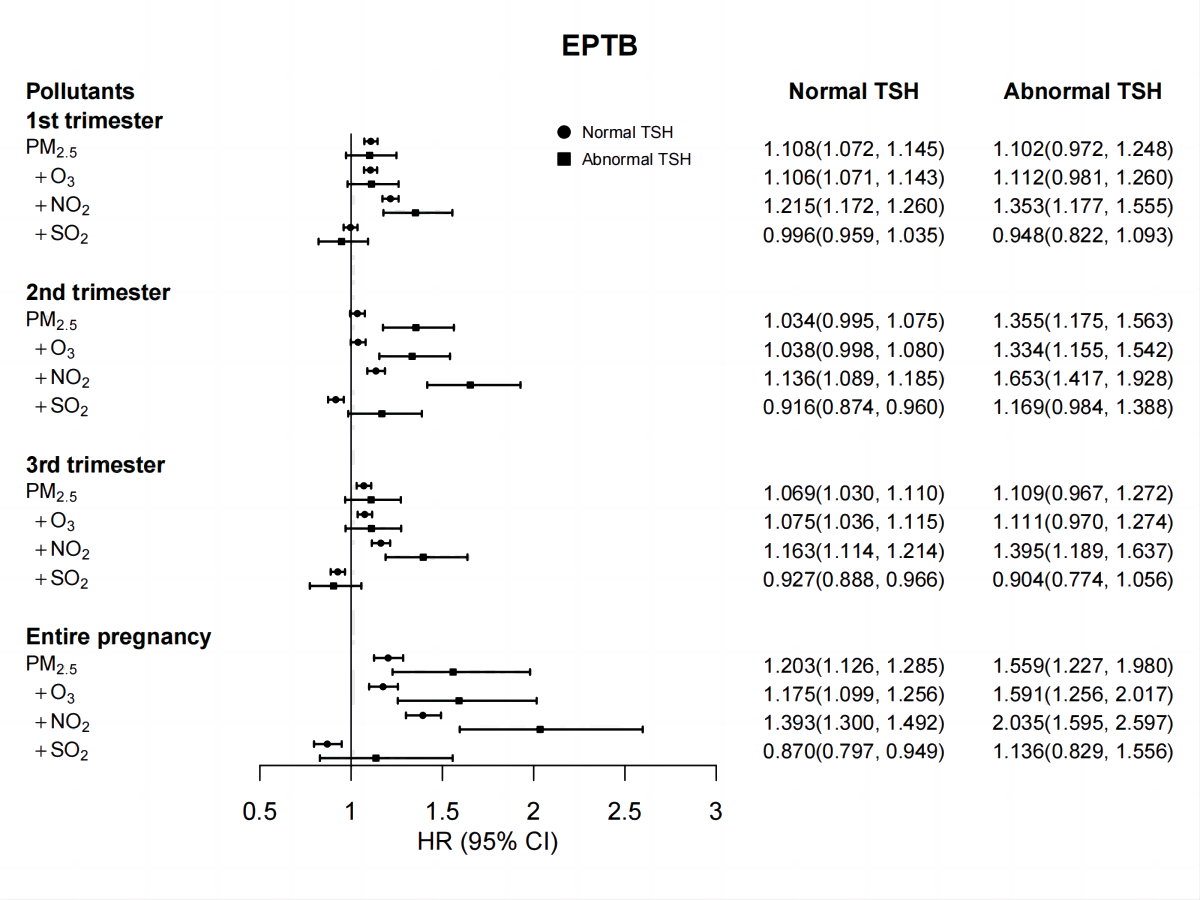

Supplement: Multimedia Appendix 6 [file publichealth-v10-e53879-s006.png]

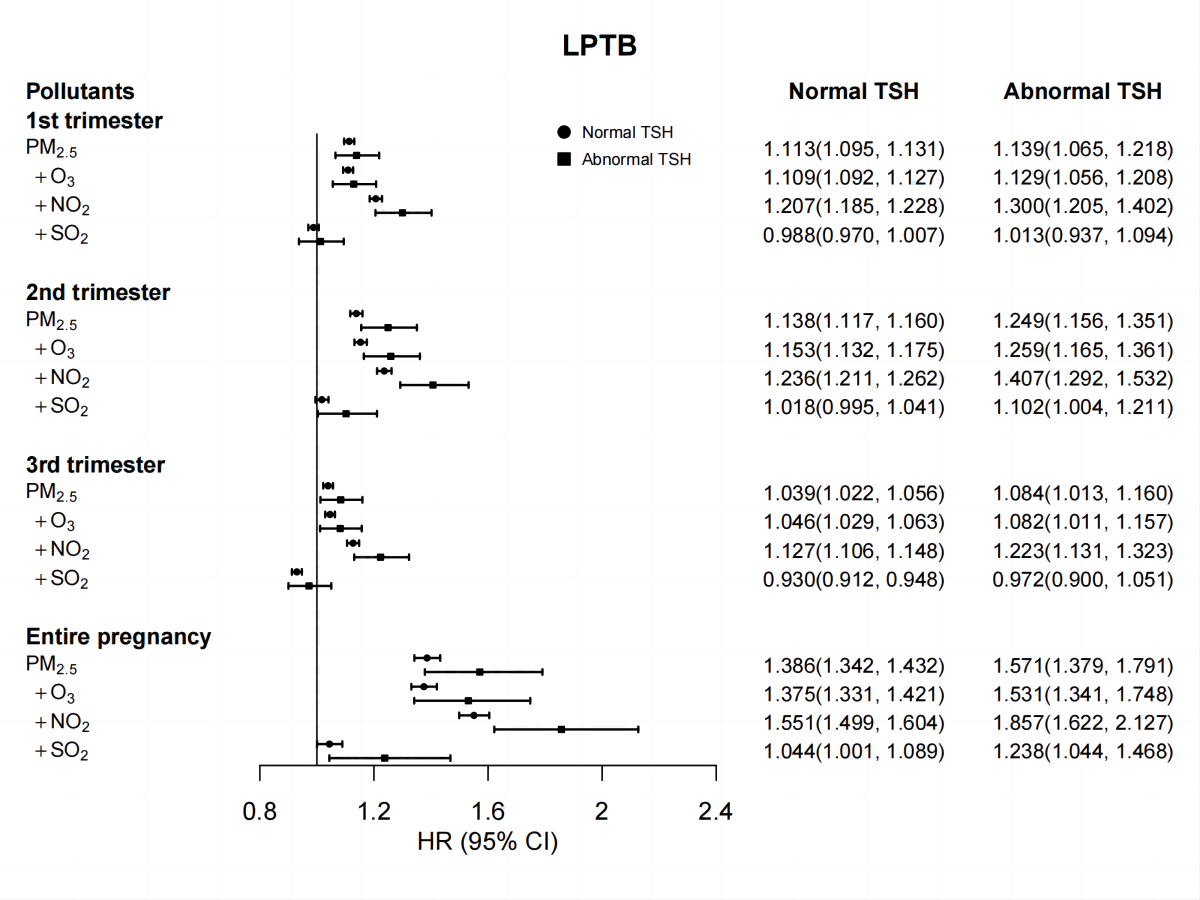

Supplement: Multimedia Appendix 7 [file publichealth-v10-e53879-s007.png]
