# Supplementary material for: Association Between Particulate Matter Exposure and Preterm Birth in Women With Abnormal Preconception Thyrotropin Levels: Large Cohort Study
Source: JMIR Public Health Surveill. 2024 Aug 2;10:e53879. doi: 10.2196/53879 (PMC11310741; doi:10.2196/53879)
Supplement: Multimedia Appendix 2 [file publichealth-v10-e53879-s002.doc]

**Table S1. Pearson correlation coefficients of air pollutants and meteorological variables during entire pregnancy.**

| Variables | PM_2.5_ | O_3_ | NO_2_ | SO_2_ | Temperature | Relative Humidity |
| --- | --- | --- | --- | --- | --- | --- |
| PM_2.5_ | 1.000 | -0.273* | 0.552* | 0.641* | -0.386* | -0.413* |
| O_3_ |  | 1.000 | -0.590* | -0.200* | 0.125* | 0.289* |
| NO_2_ |  |  | 1.000 | 0.179* | -0.268* | -0.616* |
| SO_2_ |  |  |  | 1.000 | -0.323* | -0.196* |
| Temperature |  |  |  |  | 1.000 | 0.177* |
| Relative Humidity |  |  |  |  |  | 1.000 |

^*^ P<0.05.
