# Supplementary material for: Association Between Particulate Matter Exposure and Preterm Birth in Women With Abnormal Preconception Thyrotropin Levels: Large Cohort Study
Source: JMIR Public Health Surveill. 2024 Aug 2;10:e53879. doi: 10.2196/53879 (PMC11310741; doi:10.2196/53879)
Supplement: Multimedia Appendix 3 [file publichealth-v10-e53879-s003.doc]

**Table S2. Sensitivity analyses of associations between trimester-specific PM_2.5_ exposure and risk of PTB according to maternal prepregnancy status of TSH.**

| Change in main model ^a^ | Normal TSH, HR ^b^ (95% CI) | | | |  | Abnormal TSH, HR (95% CI) | | |  |
| --- | --- | --- | --- | --- | --- | --- | --- | --- | --- |
|  | 1st trimester | 2nd trimester | 3rd trimester | Entire pregnancy |  | 1st trimester | 2nd trimester | 3rd trimester | Entire pregnancy |
| df for temperature | |  |  |  |  |  |  |  |  |
| 5 | 1.111(1.095,1.127) | 1.117(1.098,1.136) | 1.045(1.029,1.061) | 1.343(1.304,1.382) |  | 1.130(1.065,1.198) | 1.269(1.186,1.359) | 1.088(1.024,1.156) | 1.562(1.392,1.751) |
| 6 | 1.111(1.095,1.127) | 1.117(1.098,1.136) | 1.044(1.029,1.060) | 1.345(1.307,1.385) |  | 1.129(1.064,1.198) | 1.270(1.186,1.360) | 1.087(1.023,1.155) | 1.559(1.390,1.748) |
| 7 | 1.110(1.094,1.126) | 1.116(1.097,1.135) | 1.044(1.029,1.060) | 1.345(1.306,1.385) |  | 1.129(1.064,1.198) | 1.269(1.185,1.358) | 1.087(1.023,1.155) | 1.563(1.394,1.753) |
| df for RH | |  |  |  |  |  |  |  |  |
| 2 | 1.112(1.096,1.128) | 1.121(1.102,1.140) | 1.046(1.030,1.062) | 1.351(1.312,1.391) |  | 1.129(1.064,1.198) | 1.274(1.190,1.364) | 1.090(1.026,1.158) | 1.560(1.391,1.749) |
| 3 | 1.111(1.095,1.127) | 1.117(1.098,1.136) | 1.044(1.029,1.060) | 1.345(1.307,1.385) |  | 1.129(1.064,1.198) | 1.270(1.186,1.360) | 1.087(1.023,1.155) | 1.559(1.390,1.748) |
| 4 | 1.112(1.096,1.128) | 1.117(1.098,1.136) | 1.045(1.029,1.061) | 1.356(1.317,1.396) |  | 1.127(1.062,1.196) | 1.271(1.187,1.361) | 1.090(1.026,1.158) | 1.566(1.395,1.757) |

Abbreviation: PM_2.5_, particulate matter with an aerodynamic diameter of 2.5 μm or less; PTB, preterm birth; TSH, thyroid-stimulating hormone; HR, hazard ratio; CI, confidence interval.

^a^ Main Model are adjusted for maternal age, pre-pregnancy body mass index, delivery mode, newborn gender, smoking status during pregnancy, drinking status during pregnancy, mean ambient temperature and relative humidity during the pregnancy with natural cubic splines of 6 and 3 degrees of freedom respectively.

^b^ HR are based on 10 μg/m^3^ increase in PM_2.5_ exposure.
