# Supplementary material for: Association Between Particulate Matter Exposure and Preterm Birth in Women With Abnormal Preconception Thyrotropin Levels: Large Cohort Study
Source: JMIR Public Health Surveill. 2024 Aug 2;10:e53879. doi: 10.2196/53879 (PMC11310741; doi:10.2196/53879)
Supplement: Multimedia Appendix 4 [file publichealth-v10-e53879-s004.doc]

**Table S3.** Associations between trimester-specific PM_2.5_ exposure and risk of PTB according to maternal preconception TSH levels using 0.10-4.00 mIU/L as the reference range.

| PTB types | Gestational period | HR ^a^ (95% CI） | |
| --- | --- | --- | --- |
|  |  | Normal TSH ^b^ | Abnormal TSH ^c^ |
| All PTB | 1st trimester | 1.111 (1.096,1.128) | 1.127 (1.064,1.194) |
|  | 2nd trimester | 1.117 (1.098,1.136) | 1.262 (1.180,1.349) |
|  | 3rd trimester | 1.045 (1.030,1.061) | 1.081 (1.018,1.147) |
|  | Entire pregnancy | 1.346 (1.307,1.386) | 1.535 (1.372,1.717) |
| Early PTB | 1st trimester | 1.108 (1.073,1.145) | 1.099 (0.973,1.241) |
|  | 2nd trimester | 1.035 (0.995,1.076) | 1.323 (1.151,1.522) |
|  | 3rd trimester | 1.071 (1.032,1.112) | 1.114 (0.974,1.273) |
|  | Entire pregnancy | 1.204 (1.127,1.287) | 1.511 (1.196,1.908) |
| Late PTB | 1st trimester | 1.113 (1.096,1.131) | 1.137 (1.065,1.214) |
|  | 2nd trimester | 1.138 (1.117,1.160) | 1.247 (1.156,1.346) |
|  | 3rd trimester | 1.040 (1.023,1.057) | 1.074 (1.005,1.148) |
|  | Entire pregnancy | 1.387 (1.342,1.433) | 1.552 (1.365,1.764) |

Abbreviation: PM_2.5_, particulate matter with an aerodynamic diameter of 2.5 μm or less; PTB, preterm birth; TSH, thyroid-stimulating hormone; HR, hazard ratio; CI, confidence interval.

Model are adjusted for maternal age, pre-pregnancy body mass index, delivery mode, newborn gender, smoking status during pregnancy, drinking status during pregnancy, mean ambient temperature and relative humidity during the pregnancy with natural cubic splines of 6 and 3 degrees of freedom respectively.

^a^ HR are based on 10 μg/m^3^ increase in PM_2.5_ exposure.

^b^ Normal TSH: TSH >=0.1 mIU/L and TSH <= 4.0 mIU/L.

^c^ Abnormal TSH: TSH < 0.1 mIU/L or TSH > 4.0 mIU/L.
