# Supplementary material for: Association Between Particulate Matter Exposure and Preterm Birth in Women With Abnormal Preconception Thyrotropin Levels: Large Cohort Study
Source: JMIR Public Health Surveill. 2024 Aug 2;10:e53879. doi: 10.2196/53879 (PMC11310741; doi:10.2196/53879)
Supplement: Multimedia Appendix 5 [file publichealth-v10-e53879-s005.doc]

**Table S4. Associations between trimester-specific PM_2.5_ exposure and risk of PTB according to maternal preconception status of TSH by excluding participants missing baseline characteristic (N= 624,349).**

| PTB types | Gestational period | HR ^a^ (95% CI) | |
| --- | --- | --- | --- |
|  |  | Normal TSH | Abnormal TSH |
| All PTB | 1st trimester | 1.111 (1.095,1.127) | 1.126 (1.061,1.195) |
|  | 2nd trimester | 1.118 (1.099,1.137) | 1.264 (1.180,1.354) |
|  | 3rd trimester | 1.046 (1.030,1.062) | 1.084 (1.020,1.153) |
|  | Entire pregnancy | 1.348 (1.309,1.388) | 1.550 (1.381,1.739) |
| Early PTB | 1st trimester | 1.110 (1.074,1.147) | 1.103 (0.973,1.251) |
|  | 2nd trimester | 1.035 (0.995,1.076) | 1.328 (1.149,1.536) |
|  | 3rd trimester | 1.068 (1.029,1.109) | 1.090 (0.949,1.252) |
|  | Entire pregnancy | 1.207 (1.130,1.290) | 1.541 (1.210,1.962) |
| Late PTB | 1st trimester | 1.112 (1.094,1.130) | 1.134 (1.060,1.213) |
|  | 2nd trimester | 1.139 (1.118,1.161) | 1.250 (1.156,1.351) |
|  | 3rd trimester | 1.041 (1.024,1.058) | 1.084 (1.013,1.160) |
|  | Entire pregnancy | 1.389 (1.344,1.435) | 1.563 (1.370,1.782) |

Abbreviation: PM_2.5_, particulate matter with an aerodynamic diameter of 2.5 μm or less; PTB, preterm birth; TSH, thyroid-stimulating hormone; HR, hazard ratio; CI, confidence interval.

Model are adjusted for maternal age, pre-pregnancy body mass index, delivery mode, newborn gender, smoking status during pregnancy, drinking status during pregnancy, mean ambient temperature and relative humidity during the pregnancy with natural cubic splines of 6 and 3 degrees of freedom respectively.

^a^ HR are based on 10 μg/m^3^ increase in PM_2.5_ exposure.
